# A Primer to Cost-Effectiveness Analysis in Breast Cancer Imaging: A Review of the Literature

**DOI:** 10.7759/cureus.28356

**Published:** 2022-08-24

**Authors:** Joseph Waller, Kyle DeStefano, John Dempsey, Joshua Leckron, Amy Tucker, Muhammad Umair

**Affiliations:** 1 Department of Medicine, Drexel University College of Medicine, Philadelphia, USA; 2 Department of Medicine, Boston College, Boston, USA; 3 Department of Medicine, Loyola University Chicago Stritch School of Medicine, Chicago, USA; 4 Radiology, Northwestern University Feinberg School of Medicine, Chicago, USA

**Keywords:** cost-effectiveness analysis, mri, mammography, imaging, breast cancer

## Abstract

Currently, there is a multitude of methods for evaluating the costs and benefits of programs, tools, etc. While cost-benefit analysis (CBA) is commonly used, cost-effectiveness analysis (CEA) is a more appropriate method of evaluation in clinical contexts, such as radiology practices, as CEAs use units such as life years gained as opposed to money (as is the case for CBAs). This review examines CEAs performed within the past 15 years to highlight their applications and key findings in the context of medical imaging.

In total, 20 articles published between 2006 and 2022 were identified using a PubMed search for keywords including “cost-effectiveness analysis,” “breast cancer,” and “medical imaging,” with studies lacking a substantial discussion of CEA or a related topic being excluded.

CEAs have traditionally been criticized for lack of a standard methodology, despite their utility in the detection and treatment of various pathologies. Although mammography and magnetic resonance imaging (MRI) are the preferred and cost-effective imaging modalities for breast cancer, other imaging modalities, such as contrast-enhanced mammography and digital breast tomosynthesis, may be more cost-effective in the appropriate clinical context. Different combinations of mammography and MRI screenings for certain breast cancers may also prove to be more cost-effective compared to current mammography/MRI screening schedules.

While CEA has shown potential utility in estimating the costs (per unit of health gained) of different imaging tools, CEA risks ignoring important outcomes not included in the analysis and cannot address if the benefits of the imaging tool exceed its costs, as a CBA would, suggesting the need for combining several economic evaluations for a more complete understanding.

## Introduction and background

As the fastest-growing branch of medical expenditures, diagnostic imaging [1] plays a considerable role in medical decision-making and can significantly affect patient health outcomes. Breast imaging, in particular, has been contributing to this increased growth in expenditure. As new imaging modalities are developed for breast cancer screening, such as digital breast tomosynthesis (DBT) and contrast-enhanced spectral mammography (CESM), demand for a technique to assess the economic impact of these technologies increases [1]. One approach for such an assessment is the cost-effectiveness analysis (CEA), an economic evaluation of the consequences of selecting a particular imaging strategy over another [1]. CEA is a comparative analysis that allows for the comparison of medical services or interventions concerning both their costs and effectiveness. In the context of breast cancer imaging, this would be the utilization of different imaging modalities, screening frequency with these modalities, and ultimately their cost and impact on disease management. For instance, Lang et al. (2016) in their prospective, one-arm, single-institution study found that no additional breast cancers were detected by the combination of DBT and digital mammography, compared to DBT alone, suggesting the possibility of it being a stand-alone screening modality [2]. These consequences are quantified in ratios of cost per unit of effectiveness, referred to as cost-effectiveness ratios. Effectiveness is typically measured as a natural unit such as life years gained [3]. Given mutually exclusive imaging modalities, this ratio is measured marginally and known as an incremental cost-effectiveness ratio (ICER), which is a ratio of the cost of a healthcare intervention versus its effectiveness [3]. These ratios provide a way to directly measure the difference in costs and health effects between two distinct imaging modalities. In cost-utility analyses, however, effectiveness is measured as quality-adjusted life years (QALYs) [3]. This literature review will define the characteristics of CEAs in medical imaging and highlight some notable applications of cost-effective medical imaging within the past 15 years.

Zhou et al. (2018) systematically searched the literature for radiologic cost-utility studies from 1985 to 2005, revealing statistically significant methodological disparities, including distinct study perspectives, varying cost inclusion and willingness-to-pay threshold usage, and dissimilar utility measurements [4]. Moreover, Zhou et al. (2018) demonstrated that CEAs lack a standard methodology, likely resulting in contradictory interpretations of cost-effectiveness and impeding policy recommendations by government organizations [4]. Such findings led to the conclusion that a shift toward standardization of radiology CEAs should be prioritized to avoid misinterpretation and increase the comparability of the analyses. An 18-member panel on CEA in Health and Medicine was formed in 2012 to develop techniques to improve the quality of CEA, as well as the efficiency in which healthcare is delivered. The panel developed recommendations over the next several years that underwent external revision before being published in JAMA in 2016. Perhaps the biggest change in their recommendations compared to prior guidelines was the encouragement for researchers to maintain an impact inventory to keep track of all the costs associated with a healthcare tool or delivery method, which would make subsequent CEA easier by having all the costs already clearly documented [5]. Following a review of ideal studies, Goehler and Gazelle (2014) concluded that better utilization of techniques such as CEAs will require a fundamental change in how clinicians evaluate radiologic applications. Specifically, they suggest that acceptance of novel radiologic techniques should require at least a substantial change in treatment planning, and at best a significant change in patient outcomes [6]. An example of this could be DBT which, in one study, was shown to increase life years gained in women undergoing biennial breast cancer screening compared to digital mammography. Although DBT was associated with higher outright costs, it became cost-effective once the willingness to pay threshold was raised from €20,000 per life-year gained to €35,000 per life-year gained in a majority of simulations run by the MIcrosimulation SCreening ANalysis model (MISCAN) [7]. The Malmö Breast Tomosynthesis Screening Trial also showed the potential utility of using DBT as a stand-alone screening tool for breast cancer imaging which shows promise for the future [2]. This trial found significantly higher rates of breast cancer detection when using DBT (8.9 per 1,000 screens) when compared to digital mammography alone (6.3 per 1,000 scans). The increased cancer detection rate from DBT relative to digital mammography alone was 43%. Using DBT had higher recall rates, 3.8% of patients compared to 2.6% of patients who were screened by digital mammography alone. This brings about its own problems, but this number was still relatively small [2]. DBT also has higher sensitivity with dense breasts due to its three-dimensional picture reducing the adverse effects of overlapping tissue, which has been a common problem with digital mammography. Using one-view DBT for screening would also yield advantages such as lower doses of radiation and reduced compression force [2]. This highlights the importance of detecting early breast disease with these new imaging modalities. Mammography is limited by its decreased sensitivity in dense breasts, which can lead to higher costs associated with missing small cancers and the eventual more expensive treatments of advanced or metastatic disease. This has the potential to be mitigated with the newer imaging modalities mentioned above. In their analysis, Lowry et al. (2019) found that DBT could be cost-effective for biennial screening for women with dense breasts, demonstrating a possible advantage over mammography [8].

## Review

Methodology

**Figure 1:**
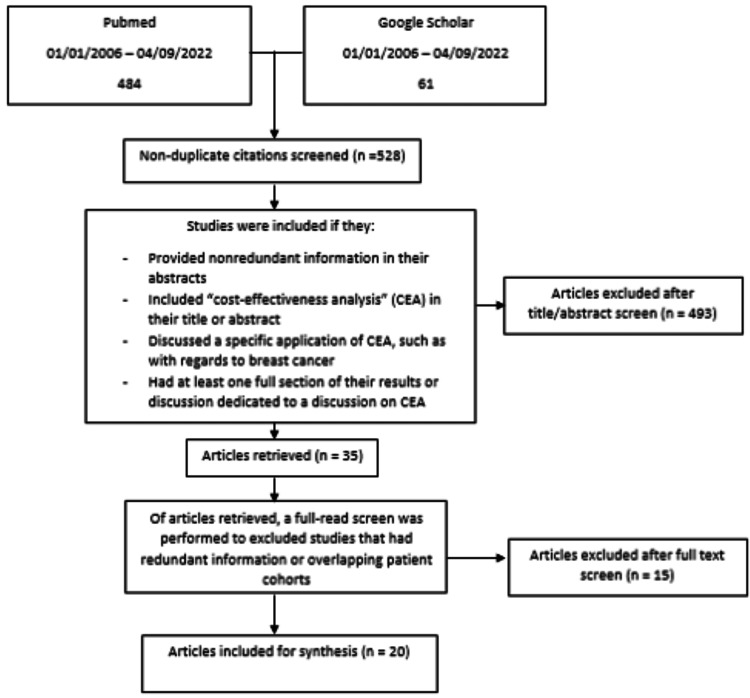
Literature search and inclusion and exclusion criteria.

The PubMed and Google Scholar databases were scanned for literature between January 1, 2006, and April 9, 2022, utilizing the application of cost-utility analyses, cost-benefit analyses, and CEAs in breast cancer imaging (Figure 1). Keywords searched in the literature included “cost-effectiveness analysis,” “medical imaging,” and “breast cancer.” In total, 484 studies were identified on PubMed and 61 on Google Scholar, yielding 528 non-duplicate studies. Of those, 493 were excluded for providing redundant information in their abstracts, not mentioning “cost-effectiveness analysis” in their title or abstract, not discussing a specific application of CEA in radiology, or not dedicating a full section of their Results or Discussion to CEA. While there is an abundance of disease processes and injuries for which radiology CEAs can prove useful, for the sake of brevity and focus, we chose to only examine imaging of breast cancers for this analysis. Ultimately, after considering the literature, we were left with 20 articles that were included in our final synthesis (summarized in Figure 1).

Results

There is a myriad of diseases for which effective diagnosis and treatment are contingent upon the appropriate use of radiologic imaging modalities and interventions. This review provides insight into the utility of CEAs in the context of imaging for breast cancer.

Breast Cancer Imaging Cost-Effectiveness as a Model

Many different imaging strategies are used to screen potential breast cancer patients. Mammography is a commonly used screening strategy, with studies suggesting up to a 30% reduction in mortality [9]. Breast magnetic resonance imaging (MRI) is an alternative imaging strategy for breast cancer screening. While it provides better sensitivity for breast cancer detection relative to mammography, it is not commonly utilized in average-risk women due to higher false-positive rates, higher costs, and less availability compared to mammograms. Conversely, CESM has shown promise as a possible breast cancer screening strategy. James et al. (2018) found through a review of the literature that when compared to MRI, CESM has fewer false positives and is also cheaper, more accessible, and actually preferred by patients [10]. Given these advantages, perhaps CESM could replace MRI in current screening strategies. Currently, in women with *breast cancer susceptibility gene* (*BRCA*) mutations, clinical guidelines suggest increasingly comprehensive screenings via MRI and digital mammography. Chubiz et al. (2013) revealed alternation between MRI and digital mammography every six months from age 30 to be a clinically effective screening approach, with an increase in QALYs as well as in costs. The strategy was found to be more cost-effective in *BRCA1 *than in *BRCA2 *carriers [11]. They also evaluated two additional strategies: the first strategy entailed alternation between MRI and digital mammography every six months from age 25 onwards, while the second strategy entailed MRI annually from age 25 with digital mammography every six months from age 30. They concluded that all three strategies increased lifetime costs and QALYs for *BRCA1- *and *BRCA2*-carrying women. Affecting these results were MRI costs and breast cancer risk estimations [11]. Other comprehensive breast cancer screening strategies for *BRCA1*- and *BRCA2*-carrying women aged 60-74 were examined in the work of Phi et al. (2019), with their work revealing that only alternating between annual mammography and MRI for *BRCA2* carriers and women with dense breasts is as cost-effective relative compared to annual mammography [12]. Currently, breast MRI utilization for mass population screening is limited by cost and availability.

The IRB-exempt Monte-Carlo population simulation model utilized by Mango et al. compared triennial MRI against annual mammography (both modalities over a 30-year period beginning at age 40) in the breast cancer screening of five million women over the age of 30 [13]. Monte-Carlo simulations involve obtaining numerous representations of stochastic processes, composed so that the simulation’s probabilistic traits match the similar values of the scenario under analysis [13]. Here, 100% screening compliance was assumed, and 2.5 million women were tested for each imaging modality of interest. To account for decreasing Medicare reimbursement rates over time, repeat simulations were performed at lower breast MRI costs ($400). Mango et al. found that it takes 24 years for aggregate MRI screening to be more cost-effective than mammography, with the cost per MRI examination ($549.71) being the largest factor affecting MRI screening program costs. However, given lower costs per MRI examination ($400, based on Medicare reimbursement trends), the MRI screening program was cost-effective relative to mammography in less than six years. The population simulation model provided conservative estimates as treatment cost savings given an earlier breast cancer diagnosis and cancer mortality productivity costs were not accounted for. The inclusion of cancer mortality productivity costs, which account for the years of economic contribution lost from the individual who dies prematurely due to cancer, allows one to account for the significant economic impact cancer morbidity and mortality has on society [14]. Table 1 summarizes the insight into the utility of cost-effectiveness analyses in the context of imaging for breast cancer.

**Table 1:** Review of CEA in imaging of breast cancer, organized by authors, imaging modalities, methods, and results. IRB = institutional review board; DBT = digital breast tomosynthesis; PET/CT = positron emission tomography/computed tomography; MBC = metastatic breast cancer; CESM = contrast-enhanced spectral mammography; CISNET = cancer intervention and surveillance modeling network; Zr = zirconium; CEA: cost-effectiveness analysis

Disease	Authors	Imaging modalities	Methods	Conclusions/Findings
Breast cancer	Chubiz et al. [11]	MRI, digital mammography	Markov Monte Carlo computer model for BRCA1- and BRCA2-carrying women	Alternating between MRI and digital mammography every six months from age 30 is more cost-effective in BRCA1 than in BRCA2 gene mutation carriers and is a clinically effective screening strategy
	Phi et al. [12]	MRI, mammography	Validated micro-simulation model	Alternating between annual mammography and MRI for BRCA2 and women with dense breasts is the only alternative strategy that is cost-effective compared to annual mammography. The other alternative strategies studied were alternating annual mammography and MRI for dense breasts only, adding annual MRI for dense breasts only, and adding annual MRI for all women
	Mango et al. [13]	MRI, mammography	IRB-exempt Monte Carlo population simulation model	Triennial MRI is more cost-effective than annual mammography after 24 years of implementation (conclusion dependent upon MRI cost)
	Lowry et al. [8]	Digital mammography, DBT	CISNET breast cancer micro-simulation models	Shifting all routine breast screening to DBT may result in a net increase in costs above commonly used cost-effectiveness thresholds (ranging from $50,000 to $150,000); however, DBT could be cost-effective at lower reimbursement rates
	Koleva-Kolarova et al. [17]	PET/CT with 18F-fluoroestradiol (FES) and ^89^Zr-trastuzumab	Extension of a previously validated computer model	PET/CT with FES and ^89^Zr-trastuzumab in first-line treatment selection for MBC patients is a potentially cost-effective treatment strategy, with even a slight increase in the sensitivity and specificity of PET/CT having a large impact on potential cost-effectiveness
	James et al. [10]	CESM, MRI	Literature comparison of CESM and breast MRI	CESM was can be cheaper to operate when compared to breast MRI. CESM offers high-resolution, low-energy images comparable to the quality seen with digital mammography. It also provides contrast-enhanced subtracted images that provided superior sensitivity and specificity compared to digital mammography, and similar sensitivity and specificity compared to breast MRI. CESM also had fewer false positives when compared to breast MRI

Approved by the FDA in 2011, DBT simultaneously minimizes soft-tissue overlap and assembles cross-sectional images of the breast. Given its superior detection rate to standard mammography, DBT could save on long-term costs by detecting more subtle disease on time, leading to lower treatment costs than treatment of advanced diseases missed on standard mammography. Lowry et al. (2019) set out to determine the long-term cost-effectiveness and health effects resulting from the transition from digital mammography to DBT as no prior research work had utilized simulation modeling for this determination. To predict the long-term economic and health outcomes of this transition, three established breast cancer microsimulation models from the Cancer Intervention and Surveillance Modeling Network (CISNET) were utilized. Microsimulation models are capable of modeling complex decisions (such as those found in medicine) while allowing one to estimate the outcome in a potentially heterogeneous sample. This is achieved through the generation of an outcome for each individual, which allows for the inclusion of variation due to individual characteristics [15]. Two screening scenarios, only DM and only DBT, were compared.

Base-case analysis revealed consistency between DBT and DM screening scenarios for breast cancer mortality and life years. ICERs for DBT ranged between $195,026 and $270,135 per QALY relative to DM, meaning the difference in incremental costs per QALY gained ranged from $195,026 to $270,135, depending on which of the three models were used. The overall transition from digital mammography to DBT increased total costs between $395,553 and $445,722 per 1,000 screening-eligible women. In an alternative scenario with additional gains for DBT sensitivity, DBT screening cost-effectiveness improved, although incremental costs per QALY remained high. DBT value improved as DBT screening examination costs decreased from the 2018 Medicare reimbursement rate [8].

Lowry et al. concluded that there was a clinically meaningful reduction in false-positive examinations, with this reduction providing an incentive for the use of DBT in routine screenings. While the transition from digital mammography to DBT could result in increased net costs given frequently utilized cost-effectiveness thresholds, the thresholds have been controversial, and cost-effectiveness may be achieved with lower reimbursement rates [8]. Potential flaws in the study include overestimation of screening utilization by the population of interest and a lack of probabilistic sensitivity analyses due to the sheer quantity of parameters for their models [16]. It is worth noting that DBT has a better detection rate than digital mammography which does have the potential to affect future costs. For example, detecting disease earlier can potentially be treated at less cost than more advanced disease that is not initially caught on standard mammography.

The presence of specific tumor receptors determines the treatment plan for non-rapidly progressive metastatic breast cancer (MBC). Koleva-Kolarova et al. (2018) investigated the cost-effectiveness of PET/CT with FES and 89Zr-trastuzumab, which can evaluate estrogen receptor and human epidermal growth factor receptor-2 expression, in first-line treatment selection for MBC patients. The strategy was shown to be potentially cost-effective, with even a small increase in the sensitivity and specificity of PET/CT having a large impact on potential cost-effectiveness. However, it remains indeterminate if this imaging strategy results in prolonged progression-free survival and overall survival with maintained quality of life. This is also a controversial point as PET/CT has several limitations. These limitations include the fact that smaller metastasis are often below the PET threshold, it is not always helpful to detect smaller-volume disease, and lobular breast carcinomas and its metastasis, in particular, can be underestimated or may not be detected at all. The cost-effectiveness was determined by extending and adapting a previously validated computer model. Many of the limitations in the study arose from the lack of clinical data from randomized controlled trials and observational studies. Limitations include lack of consideration of complications from invasive pathology tests, the receptor heterogeneity displayed by much of the patient population, and the assumption of one number for the specificity and sensitivity of the imaging strategies, given the fact that sensitivity and specificity are largely dependent on the expression levels of the target, the instrumentation and the tracer used, among others [17].

Discussion

**Figure 2:**
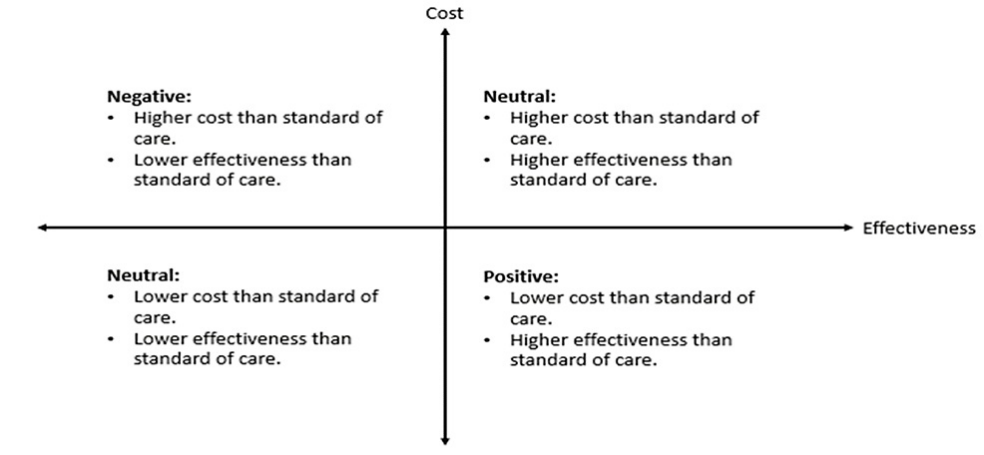
Cost-effectiveness plane.

CEAs can be a useful tool for comparing multiple different imaging techniques to determine whether the cost associated with a given technique taken together with its clinical benefits is superior, equal to, or inferior to other available techniques (such classification is made easier through the use of a CEA plane, such as the one seen in Figure 2).

Various thresholds and measurements can be used to facilitate the economic analysis of a given imaging modality, such as QALYS, ICERs, and willingness-to-pay thresholds. Willingness-to-pay thresholds were an important consideration in several of the studies examined in this review. These refer to the maximum amount one is willing to pay for a given service [18]. Therefore, essentially, willingness to pay is a determinant of fair pricing from the point of view of the consumer, more like an estimate of the fair pricing for medical services from a patient’s perspective for our purposes [19]. Willingness-to-pay thresholds are of critical importance as the economic burden placed upon an individual by the cost of a given treatment or imaging strategy may become great enough that they no longer consider the monetary cost worth the health benefits derived from the service in question. One guideline for identifying CEAs discussed a case study of a hospital library’s CEA and determined that a true CEA must provide evidence for and a suitable unit of measurement of the effectiveness, as well as recognition of all the relevant costs and a study question that incorporates both the costs and effectiveness [20].

CEAs do not always point toward a definitive imaging approach, as is demonstrated by several of the studies in this review. A key determinant here is the fact that a CEA compares the financial feasibility of different tests and by no means is a predictor of the clinical and statistical efficacy/accuracy of a test, which can be more important determinants of choosing between different tests in a clinical setting. The work performed by Phi et al. is an example of how certain imaging strategies can have both clinical and financial advantages and disadvantages, a situation wherein the advantages should be examined in the context of a particular patient to determine if a given radiologic technique is appropriate given available resources and the goals of treatment [11]. In addition to knowing that in the majority of cases CEAs act as further guidance rather than a definitive tool for deciding which imaging technique to utilize, it is important to discuss the limitations of the data and simulations used in a given CEA.

Some projected outcomes, such as the results of the Lowry et al. study examining the benefits of DBT, are based on currently observed screening patterns, and, therefore, may reflect underestimated mortality benefits relative to ideal conditions [15]. Moreover, such estimates must make assumptions regarding screening utilization and may involve over or underestimations of screening in the population of interest. The Lowry et al. study also serves as a prime example of how controversial factors such as reimbursement rates (a factor also discussed in the work of Mango et al. on breast cancer) inevitably impact the cost associated with a particular imaging modality. One potential strategy for overcoming reimbursement-dependent outcome variation is to run repeat simulations that differ in the reimbursement rates used (with such a strategy being employed by Mango et al. to account for decreasing Medicare reimbursement rates). Other factors worthy of consideration are the method by which one decides expected outcome frequencies for each variable modeled, the country/location from which data were gathered, and, when applicable, the level of screening compliance assumed.

Limitations

This is a small sample of some key studies encompassing only breast cancer imaging as a model modality. While this review can certainly serve as a primer to one’s education on CEAs in radiology, it is by no means an exhaustive examination of the field, and the methods presented and critiqued do not cover all methods utilized by those performing radiologic imaging CEAs. Additionally, most of the techniques studied here are applied to diagnostic imaging, limiting the utility of the study for those interested purely in cost-effectiveness in interventional radiology.

## Conclusions

CEAs provide insight into many different alternative imaging strategies but can be greatly limited by the conditions set by both the constructed models and the researchers themselves.

CEA compares the financial feasibility of different tests and by no means is a predictor of the clinical and statistical efficacy/accuracy of a test, which can be more important determinants of choosing between different tests in a clinical setting. In addition to knowing that in most cases CEAs act as further guidance rather than a definitive tool for deciding which imaging technique to utilize, it is important to discuss the limitations of the data and simulations used in each CEA.

Understanding the assumptions and limitations of a given CEA is crucial to determining how applicable the results of the study are to one’s own practice.

CEA has shown potential utility in estimating the costs (per unit of health gained) of different imaging tools and emphasizes improving patient outcomes. Despite these benefits, CEA risks ignoring important outcomes and externalities not included in the analysis. This is because CEA almost always includes assumptions, and it is unlikely to have a measure of everything necessary for a comprehensive analysis. This suggests the need for combining several economic evaluations for a more complete picture of the comparisons between differing breast screening modalities and protocols, including their cost, health benefits, sensitivity, specificity, and recall rates.
